# Short communication of the challenges and recommendations about the unreliable control of COVID19 in Yemen

**DOI:** 10.1016/j.amsu.2022.103876

**Published:** 2022-05-29

**Authors:** Mohammad Badr Almoshantaf, Asma'a Munasar Alsubari, Haya Bassam Al-Kubati, Karam R. Motawea, Eman Mohammed sharif Ahmed, Sheikh Mohd Saleem, Sheikh Shoib, Mohammad Mehedi Hasan

**Affiliations:** a Neurosurgery Department, Ibn Al-Nafees Hospital, Damascus, Syria; b Faculty of Medicine, Sana'a University, Sana'a, Yemen; c Faculty of Medicine, 21 September University, Sana'a, Yemen; d Faculty of Medicine, Alexandria University, Egypt; e NILE VALLY UNIVERSITY, Atbra, Sudan; f Independent Health Researcher, J&K, India; g JLNM Hospital, Rainawari, Srinagar, Directorate of Health Services, J&K, India; h Department of Biochemistry and Molecular Biology, Faculty of Life Science, Mawlana Bhashani Science and Technology University, Tangail, Bangladesh

Coronavirus is one of the most present-day infections that has produced a worldwide health catastrophe and substantially impacted the global population. COVID-19 virus has continued to spread worldwide. The global response has been an unpleasant combination of despair, exhaustion, and déjà vu. Almost two years into a pandemic that has claimed over five million lives and affected billions more, people all over the world are struggling to find the energy to write another stage in the development. As no definitive therapy or vaccination can completely eradicate the disease, COVID-19 is still a significant concern [1].

Yemen's civil war began in 2014, when Houthi insurgents—Shiite rebels with ties to Iran and a history of rising up against the Sunni government—took control of Sana'a, Yemen's capital and largest city, demanding lower fuel costs and a new administration. Yemen had the most onerous burden among other countries due to the circumstances it had been living in since the commencement of the war in 2011, the rise of the armed conflict, and the five-year embargo. Even before the COVID-19 pandemic, Yemenis were forced to fend for themselves and confronted numerous challenges such as famine, the continuous war, infections, diseases, and a lack of equipment. Yemen continues to be one of the world's most dire humanitarian crises. Prolonged armed conflict, widespread economic collapse, and overburdened national systems and services have left 70% of the total population in need of humanitarian assistance, including 11.3 million children. The protracted situation has had a negative impact on children's health and nutrition: nearly 400,000 children are severely malnourished, and 2.3 million are acutely malnourished [2]. The COVID-19 pandemic strained an already frail health system and exacerbated children's, adolescents', and women's underlying protection and gender-related vulnerabilities. Because of the uprisings that afflicted Libya, Syria, and Yemen, they are on the verge of collapsing due to the spread of the COVID-19 [3]. The gaps created by wars and political conflicts have weakened the health system and created further barriers to control the virus's spread. It's important to mention that the International Human Rights Laws (IHRL) is alerting and giving directions about the importance of caring for human life and that citizens' exposure to the COVID = 19 virus is an indirect war [4]. Yemen proclaimed the first instance of an epidemic on the 10th of April 2020. Because it was the last country globally to declare an epidemic, many people were concerned about the epidemic's disastrous occurrences in Yemen owing to the country's humanitarian crises. During the first two months, some researchers' reports have summarised the situation and predicted relatively bad outcomes. Between February 18 and June 5, 2020, they confirmed 469 laboratory groups, 552 probable cases, and 55 suspected cases. The confirmed patients ranged from one year to ninety years, with 75 of the confirmed cases being males. They also recorded 111 deaths among those infected with the illness and confirmed infection, with an average age of 53 years for those who died, ranging from 14 to 88 years, with 63% of deaths, or 70 deaths, happening in those under the age of 60. Two hundred sixty-eight people, or 57%, were infected, of whom 95 died in hospital.

There are extremely few completely operational COVID-19 treatment centres in Yemen. In other treatment centres, health workers frequently do not feel comfortable working without the necessary protective equipment, and fear of stigma prevents admission to the few functional centres. Because of these constraints and limited resources, mild and moderate cases were not recorded, cared for, or transported to health institutions. Consequently, statistics and reports were based on severe and incurable cases. As they could not reach the northern portion of the nation to survey or further evaluate the circumstances, this study only included the southern and eastern parts of the country, which account for 31% of Yemen's total population [5]. Testing COV1D-19 in Yemen was limited to only four out of six entire major public health laboratories in Sana'a, Aden, Taiz, and Mukalla governorates. Those laboratories were equipped to have the capacity to test for COVID-19 using RT-PCR under World Health Organization (WHO.

## Sources of funding

This research did not receive any specific grant from funding agencies in the public, commercial, or not-for-profit sectors.

## Ethical approval

N/A.

## Consent

N/A.

## Author contribution

All authors have participated in writing and reviewing the manuscript.

## Registration of research studies

Not applicable.

## Guarantor

Mohammad Badr Almoshantaf.

## Declaration of competing interest

All authors declare no conflict of interest.

## References

[1] Dhabaan G., Chahin A., Buhaish A., Shorman M. (2020). COVID-19 pandemic in Yemen: a questionnaire based survey, what do we know?. J Infect Dev Ctries.

[2] Unicef Yemen appeal [internet]. UNICEF. 2021 [cited 2022 feb 27]. https://www.unicef.org/appeals/yemen.

[3] Daw M.A. (2021). https://www.frontiersin.org/article/10.3389/fpubh.2021.667364.

[4] Van Hout M.C., Wells J.S.G. (2021). The right to health, public health and COVID-19: a discourse on the importance of the enforcement of humanitarian and human rights law in conflict settings for the future management of zoonotic pandemic diseases. Publ. Health.

[5] Al-Waleedi A.A., Naiene J.D., Thabet A.A.K. (2020). The first 2 months of the SARS-CoV-2 epidemic in Yemen: analysis of the surveillance data. PLoS One.

